# Validity of Velocity Measurements of a Motorized Resistance Device During Change of Direction

**DOI:** 10.3389/fphys.2022.824606

**Published:** 2022-02-24

**Authors:** Ola Eriksrud, Fredrik Ahlbeck, Damian Harper, Øyvind Gløersen

**Affiliations:** ^1^Department of Physical Performance, Norwegian School of Sport Sciences, Oslo, Norway; ^2^School of Sport and Health Sciences, Institute of Coaching and Performance, University of Central Lancashire, Preston, United Kingdom

**Keywords:** kinematic analysis, phase analysis, performance, motorized resistance technology, acceleration, deceleration

## Abstract

The aim of this study was to determine validity of velocity measurements of a motorized resistance device (MRD) during change of direction (CoD). Eight male (age: 22.1 ± 4.2 yrs; weight: 83.3 ± 17.1 kg; height: 181.6 ± 12.6 cm) and three female participants (age: 21.7 ± 1.5 yrs; mass: 69.7 ± 2.4 kg; height: 167.0 ± 3.6 cm) completed the modified 505 CoD test (m505) with turning off the left and right foot while exposed to external loads (3, 6, and 9 kg) provided by the MRD. Three-dimensional kinematic data were measured (200 Hz) for all tests using a full-body marker set with an additional marker placed on the pulley used to attach the carabiner (CAR) at the end of the line from the MRD to the participant. Average velocity of overall center of mass (COM_vel_), pelvis (COM_pelvis_vel_), and pulley (CAR_vel_) was then calculated and compared to the velocity measured by MRD (MRD_vel_) in 0.5 s intervals 1.5 s before and after CoD. Average velocities from these intervals were then compared using correlational, Bland–Altman analysis, coefficient of variation (CV), and statistical parametric mapping (SPM). Mostly, excellent correlations were observed and ranged from 0.93 to 1.00, 0.53 to 1.00 and 0.93 to 1.00 for the 3, 6, and 9 kg load conditions, respectively. CV values ranged from 0.3 to 3.2%, 0.8 to 4.3%, and 1.5 to 7.7% for the CAR_vel_, COM_pelvis_vel_, and COM_vel_ comparisons, respectively. The biases for CAR_vel_ comparisons ranged from −0.027 to 0.05 m/s, −0.246 to 0.128 m/s and −0.486 to 0.082 m/s across all load conditions and time intervals for the CAR_vel,_ COM_pelvis_vel_, and COM_vel_ comparisons, respectively. SPM analysis indicated significant differences between MRD_vel_ and COM_vel_ and COM_pelvis_vel_ over short time periods during the CoD, but no difference between MRD_vel_ and CAR_vel_. The velocity measurements obtained by a MRD during a m505 test are valid as low biases, low CV’s, and high correlations are observed for the MRD_vel_ to CAR_vel_ comparison. As single points of measurement (i.e., laser) has been proven useful to assess other athletic tasks (i.e., sprint running), the single point CAR_vel_ comparison is an appropriate comparison for validating MRD_vel_ measurements during the m505 test.

## Introduction

Change of direction (CoD) is an important skill in multi-directional sports and identified as an essential component underpinning agility where athletes are required to make quick and precise CoD maneuvers to enable successful tactical and technical outcomes. CoD has been defined as “the skills and abilities needed to change movement direction, velocity, or modes” (DeWeese and Nimphius, 2016) where the ability to co-ordinate force application during each phase of CoD including: (1) initial acceleration, (2) deceleration, (3) turn, and (4) re-acceleration is important (Dos’Santos et al., 2017). In invasion sports, CoD ability is important for penetrating defensive lines (Wheeler et al., 2010; Fox et al., 2014; Mohamad Zahidi and Ismail, 2018), creating goal scoring opportunities (Faude et al., 2012), talent identification (Gil et al., 2007), discriminating between levels of performance (Reilly et al., 2000), and for draft selection in the National Football League (McGee and Burkett, 2003). Considering the importance of CoD in multi-directional sports it is necessary to have valid tests to quantify this quality.

Currently, a plethora of tests are used to quantify CoD based on different movement patterns (i.e., sprint and side shuffle), angle of turn, number of turns, and duration (Nimphius et al., 2017). Such differences make comparisons between tests difficult as CoD is a task-specific skill based on angle of turn and entry velocity (Nimphius et al., 2017; Dos’Santos et al., 2018). In most CoD tests, the overall time has commonly been used as the primary outcome variable to quantify CoD ability. However, a number of problems have been raised with this primary outcome measure. Firstly, longer tests might not be representative of CoD, but rather anaerobic capacity and linear sprint ability (Vescovi and McGuigan, 2008). Secondly, even in shorter tests, such as the modified 505 (m505) which consists of two 5 m sprints with a 180° turn, superior sprint capacity can still mask CoD ability (DeWeese and Nimphius, 2016; Nimphius et al., 2017). As a result, indirect measures, such as the COD deficit, have been developed in an attempt to better quantify CoD ability by isolating the CoD component (Nimphius et al., 2016).

Based on the above shortcomings of current CoD testing approaches, it has been advocated that practitioners should aim to quantify an athletes center of mass velocity (COM_vel_) during CoD actions (Nimphius et al., 2016). To obtain such measurements in a laboratory setting (i.e., motion capture) and calculate COM velocity is not difficult, but it is not practical and in many cases not feasible for coaches and other practitioners in the applied setting. Rather, field-based technologies, such as photocells (Buchheit et al., 2012; Hader et al., 2015; Nimphius et al., 2017) global navigation satellite systems (GNSS) and local positioning systems (LPS) technologies (Meylan et al., 2017; Luteberget and Gilgien, 2020) and laser (Hader et al., 2015) have been used to assess CoD ability. Photocells are commonly used to obtain overall time of the CoD test, but do not provide phase-specific information (Buchheit et al., 2012; Hader et al., 2015). Furthermore, most GNSS or LPS measurements have limited validity and reliability for short CoD tests (Buchheit et al., 2014; Meylan et al., 2017; Luteberget and Gilgien, 2020) due to limitations in sampling frequency and position measurement accuracy. In a recent study, Hader and co-workers designed a football-specific field test based on two synchronized laser systems. This to explore phase-specific information with different turn angles, which in turn could have practical implications if either initial acceleration to deceleration, re-acceleration, or both should be targeted in training (Hader et al., 2015). Such phase-specific information is important considering that some athletes have been shown to self-pace their run-up (initial acceleration-to-deceleration phase) based on the demand of the CoD (Nimphius et al., 2013). This is also in agreement with field-based observations of the authors and colleagues. Without continuous direct measurements of athlete movement during a CoD test such phase-specific information cannot be obtained.

Recently, development of new technologies may provide scientists, practitioners, and coaches with an opportunity to obtain more detailed information about an athletes CoD ability in both lab- and field-based environments. Motorized resistance technology can be applied to CoD testing to provide continuous velocity measurement of the athlete and thereby provide phase-specific information on CoD test performance, while at the same time prescribe horizontal load. With valid phase-specific velocity measurements obtained throughout CoD more detailed insights can be obtained to direct CoD training prescription. For example, more detailed insights into the deceleration phase as introduced by Harper and co-workers could be explored (Harper et al., 2020). Additionally, since an athletes momentum could have a significant effect on CoD performance (Fernandes et al., 2020), continuous velocity obtained with motorized resistance device (MRD) could also enable exploration of an athletes change in momentum capabilities throughout CoD as previously advocated by Nimphius et al. (2017). Motorized resistance technology has recently been applied for both linear sprint testing and training purposes (Rakovic et al., 2018, 2022; Lahti et al., 2020), but to the authors knowledge not currently to CoD testing. Accordingly, the aim of this study was to assess validity of the velocity measurements obtained with a MRD to marker-based three-dimensional motion capture data during the m505 test under different loaded conditions. Specifically, we hypothesized that velocity measured by the MRD would be in close agreement with a marker placed at the MRD attachment point on the athlete, but that there would be modest biases compared to the segmental velocity and overall COM velocity.

## Materials and Methods

### Subjects

Eight male (age: 22.1 ± 4.2 yrs; mass: 83.3 ± 17.1 kg; height: 181.6 ± 12.6 cm) and three female participants (age: 21.7 ± 1.5 yrs; mass: 69.7 ± 2.4 kg; height: 167.0 ± 3.6 cm) with experience in ball sports [soccer (*n* = 2), basketball (*n* = 4), and handball (*n* = 3), tennis (*n* = 1) and floorball (*n* = 1)] completed the study. Inclusion criteria were familiar with ball sports CoD movements and no musculoskeletal or neurological injury within the past 6 months limiting sports participation for more than 1 week. The study was approved by the local Ethical committee and the National Data Protection Agency for Research (ref number: 148213) and conducted in accordance with the Declaration of Helsinki. Prior to participation all participants, or legal guardian, provided a written informed consent after being given detailed verbal and written explanation of the purpose, procedures, and risks associated with participation.

### Procedures

All participants had one familiarization session prior to the test session as recommended for the modified 505 test (m505) (Barber et al., 2016). Anthropometric measurements (height and weight) were obtained prior to a standardized warm-up (jogging, forward and backward, side shuffle, lower extremity mobility exercises, jumps, sprint, and two unloaded m505 tests on each foot) and lasted approximately 15 min. The same warm-up was used for both familiarization and test session.

Testing took place in the biomechanics laboratory at the Norwegian School of Sport Sciences where subjects performed two successful repetitions of the m505 test with turns off both the left and right foot. Procedures have been described in detail previously (Draper and Lancaster, 1985; Taylor et al., 2019) but summarized here for clarity as it was performed under externally loaded conditions provided by MRD. For all tests, the subject started with a two-point start at a 5 m mark (tape) from the center of the second force plate (tape mark on sides). The fiber cord from the MRD was attached to the subject using a carabiner onto a pulley (Cyclone 52; Purmotion, United States), which in turn was attached to a belt with two carabiners (1080 Vest; 1080 MAP AS, Oslo, Norway). When turning off the left foot, the carabiners were attached over the right hip and for right foot turns vice versa. This to ensure that the fiber cord from the MRD was not in conflict with the CoD movement. As the initial acceleration was toward the MRD this portion of the test was assisted with a greater demand placed on the deceleration and re-acceleration. A successful trial was defined as full effort with the penultimate and final foot contact hitting the floor-mounted force plates. The external load protocol was tested in the order of 3, 9, and 6 kg with two successful turns off the left before the right foot for each load condition. A 2-min rest period was given between trials.

### Equipment

Three-dimensional kinematic data were measured (200 Hz) using 16 Oqus (eight 700+ series (resolution 4,096 × 3,072 pixels), eight 400 series (resolution 1,712 × 1,696 pixels), Qualisys AB, Gothenburg, Sweden) of a full-body marker set (63 markers) and one marker placed on the pulley used to attach the carabiner (CAR) at the end of the line from the MRD. The system was calibrated according to the manufacturers recommendations. Calibration accuracy (standard deviation of calibration wand length) was <3 mm for all trials. The approximate recording volume was 5 m (length and width) and 2 m (height), and the laboratory co-ordinate frame was defined so that the y-axis was aligned with the initial running direction.

**Figure 1:**
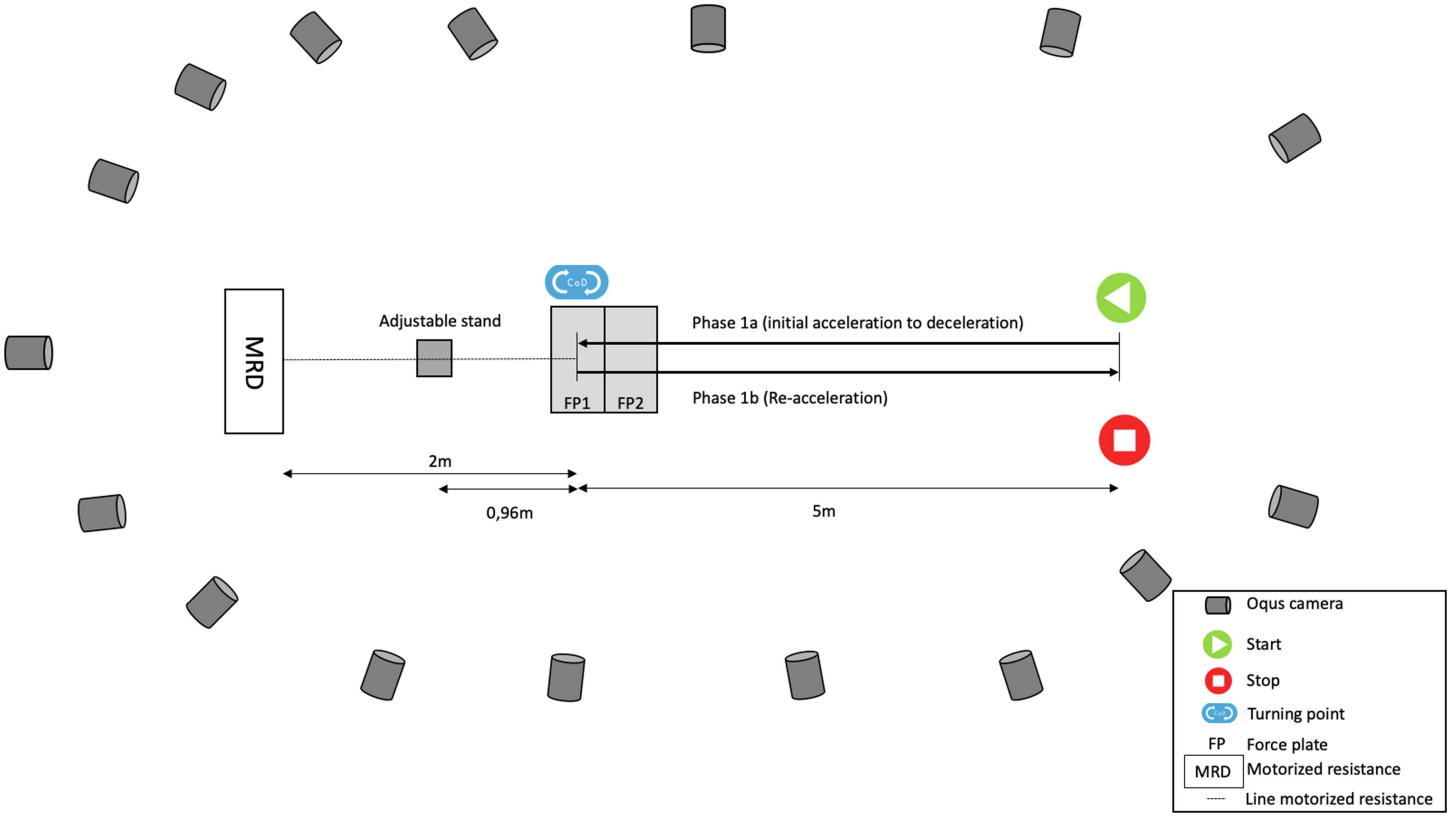
Laboratory set up illustrating placement of Oqus cameras, motorized resistance device, force plates and start and end positions of m505 test.

A portable MRD (1080 Sprint; 1080 Motion, Lidingö, Sweden) was used to provide external resistance and measuring time and position at 333 Hz. The MRD measurements were down-sampled to the motion capture sampling frequency (200 Hz) using linear interpolation. MRD velocity (MRD_vel_) was calculated as the first time derivative of position data. The 1080 Sprint (MRD) has a servo motor (2000 RPM OMRON G5 Series Motor; OMRON Corp., Kyoto, Japan) that is attached to a carbon fiber spool around which a fiber cord is wrapped. The MRD was positioned on a table 2 m behind the force plates and perpendicular to m505 running directions to allow for the m505 test to be performed along the global y-axis. The fiber cord was also passed through a feeder on an adjustable stand (0.96 m behind the force plate) used to adjust line to hip height (greater trochanter) of each subject. The loads used were 3, 6, and 9 kg. The auto start function of the MRD was used (onset of measurement with speed >0.2 m/s) (Rakovic et al., 2022). See Figure 1 for a description of laboratory set up.

### Data Analysis

Marker locations were registered in a static standing trial in order to determine the static calibration of the kinematic model. Local co-ordinate systems for the different segments were created based upon established recommendations from the International Society of Biomechanics (Wu et al., 2002, 2005). Specifically, the following segments were created: (1) foot based on the recommendation of Hamill and co-workers (Robertson et al., 2014), (2) leg (Wu et al., 2002), (3) thigh using the prediction approach to calculate the hip joint center (Bell et al., 1989; Wu et al., 2002), (4) pelvis (Wu et al., 2002; Leardini et al., 2011), (5) thorax (Wu et al., 2005; Leardini et al., 2011), and (6) upper arm, forearm, and hand (Wu et al., 2005) (Visual 3D, C-Motion Inc., Rockville, MD, United States). The marker position data were filtered with a second-order bi-directional Butterworth low pass filter with a cutoff frequency of 15 Hz. Then, overall center of mass (COM) and pelvis (COM_pelvis_) were calculated from segmental COM data. Velocity and acceleration for CAR, COM, and COM_pelvis_ were then calculated as first (CAR_vel_, COM_vel_, and COM_pelvis_vel_) and second time derivative of position data (CAR_acc_, COM_acc_, and COM_pelvis_acc_), respectively. Only the y components of these variables were used, since the y-axis was aligned with the m505 running direction. The motion capture data were time-aligned with the MRD data by cross-correlating the acceleration measurements of both systems. This was done using Matlab R2021a (The MathWorks, Inc., Natick, United States) by finding the peak cross-correlation between CAR_acc_ and MRD_acc_, after low pass filtering the CAR position with a low pass filter equivalent to that built into the MRD (Tustin filter with filter coefficient 0.04 s).

As different definitions of time of CoD are used (Sayers, 2015; Clarke et al., 2020) or not defined (Hader et al., 2015), we defined time of CoD based on countermovement jump definitions (McMahon et al., 2018). Specifically, time of CoD was defined as the time when direction of MRD_vel_ changed (MRD_vel_COD_). From this, phase 1a was defined from the onset of measurement to MRD_vel_COD_, while phase 1b was defined from to MRD_vel_COD_ to start position. Then, velocity (MRD_vel_, CAR_vel_, COM_vel_, and COM_pelvis_vel_) at 0.1 s time intervals before (−) and (+) after MRD_vel_COD_ were defined. Also, average velocity for 0.1, 0.5, 1.0, and 1.5 s intervals before (−) and after (+) MRD_vel_COD_ were also calculated for all outcome variables (MRD_vel_, CAR_vel_, COM_vel_ and COM_pelvis_vel_). In addition, the following performance outcome measurements were obtained from the MRD for the m505 test: total time (m505_time_), total distance (m505_dist_), time phase 1a (m505_1a_time_), distance 1a (m505_1a_dist_), average velocity phase 1a (m505_1a_avgvel_), time phase 1b (m505_1b_time_), distance 1b (m505_1b_dist_), and average velocity phase 1b (m505_1b_avgvel_).

### Statistical Analysis

Descriptive statistics [mean and standard deviation (SD)] were calculated in Excel version 14.4.8 (Microsoft Corp., Redmond, WA, United States). All other statistical tests were done using IBM SPSS version 21.0 (IBM, Armonk, NY, United States). Normality of the data was assessed using Shapiro–Wilk’s test (*α* = 0.05). The criterion-related (concurrent) validity of the MRD was determined by comparing MRD_vel_ to CAR_vel_, COM_vel_, and COM_pelvis_vel_ measurements during different time intervals using correlational analysis, including Pearson product-moment correlation (*r*) and Spearman rank-order correlation (*ρ*), for normal and non-normal distribution, respectively. Interpretation of correlation coefficients was done according to the guidelines of Hopkins (Hopkins, 2011) as follows: impractical (<0.45), very poor (0.45–0.70), poor (0.70–0.85), good (0.85–0.95), very good (0.95–0.995), and excellent (>0.995). Coefficient of variation (CV %) was calculated using a custom Excel spreadsheet (Hopkins, 2015). Bland–Altman method was employed to determine bias and limits of agreement for the different time intervals as defined previously. Furthermore, statistical parametric mapping (SPM; Pataky et al., 2015) using paired *t*-tests (SPM(*t*); *α* = 0.05; two-tailed) was used to determine if velocity from MRD_vel_ was different from kinematic data (CAR_vel_, COM_pelvis_vel_, and COM_vel_) 1 s before (−) and after (+) CoD for the different load conditions.

## Results

A total of 40, 40, and 44 tests were analyzed for the 3, 6, and 9 kg loaded conditions, respectively. Performance on the m505 tests, average left and right turns, ranged from 3.26 to 3.52 s for the different load conditions. Phase-specific times ranged 1.77 to 1.83 s and 1.47 to 1.69 s for phase 1a and 1b, respectively, for the different load conditions (Table 1).

**Table 1:** m505 test results for the different loaded conditions.

Performance variable	3 kg		6 kg		9 kg	
	*M*	SD	*M*	SD	*M*	SD
m505_time_ (s)	3.26	0.29	3.32	0.35	3.52	0.33
m505_dist_ (m)	9.71	0.33	9.81	0.42	10.02	0.45
m505_1a_time_ (s)	1.78	0.15	1.77	0.22	1.83	0.15
m505_1a_dist_ (m)	4.86	0.16	4.91	0.21	5.01	0.23
m505_1a_avgvel_ (m/s)	2.76	0.17	2.83	0.22	2.78	0.16
m505_1b_time_ (s)	1.47	0.16	1.56	0.16	1.69	0.21
m505_1b_dist_ (m)	4.86	0.16	4.91	0.21	5.01	0.23
m505_1b_avgvel_ (m/s)	3.24	0.27	3.10	0.25	2.94	0.27

M, mean; SD, standard deviation; m505_time_, total time; m505_dist_, total distance; m505_1a_time_, time phase 1a; m505_1a_dist_, distance 1a; m505_1a_avgvel_, average velocity phase 1a; m505_1b_time_, time phase 1b; m505_1b_dist_, distance 1b; m505_1b_avgvel_, average velocity phase 1b.

**Figure 2:**
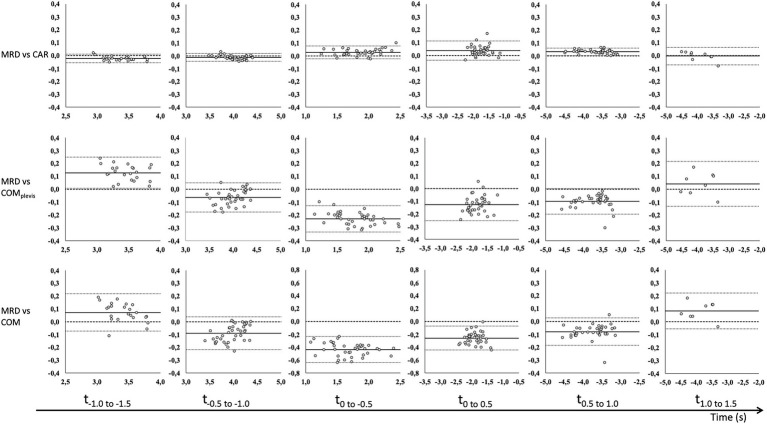
Bland–Altman analysis of average velocity for 0.5 s time intervals before (−) and after (+) CoD (horizontal axis) for the MRD to CAR (top row), COM_pelvis_ (middle row), and COM (bottom row) for 3 kg external load. Bland–Altman plots [y-axis: difference in velocity (m/s) and x-axis: average velocity (m/s)] for all trials with fixed bias (full line) with 95% confidence interval (dotted line) and agreement (dashed line).

**Figure 3:**
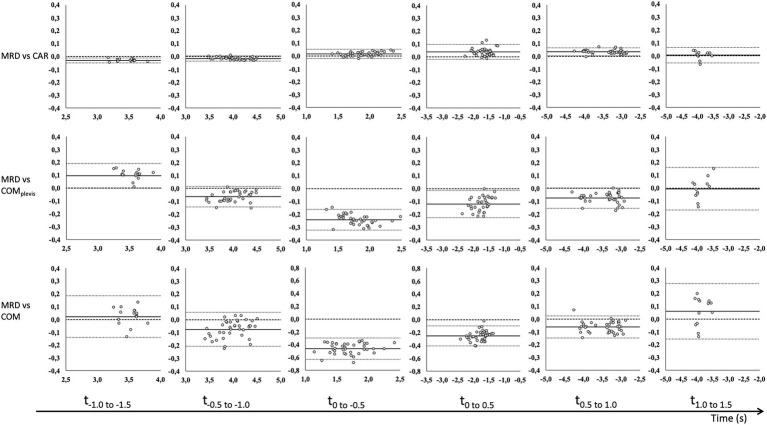
Bland–Altman analysis of average velocity for 0.5 s time intervals before (−) and after (+) CoD (horizontal axis) for the MRD to CAR (top row), COM_pelvis_ (middle row) and COM (bottom row) for 6 kg external load. Bland–Altman plots [y-axis: difference in velocity (m/s) and x-axis: average velocity (m/s)] for all trials with fixed bias (full line) with 95% confidence interval (dotted line) and agreement (dashed line).

**Figure 4:**
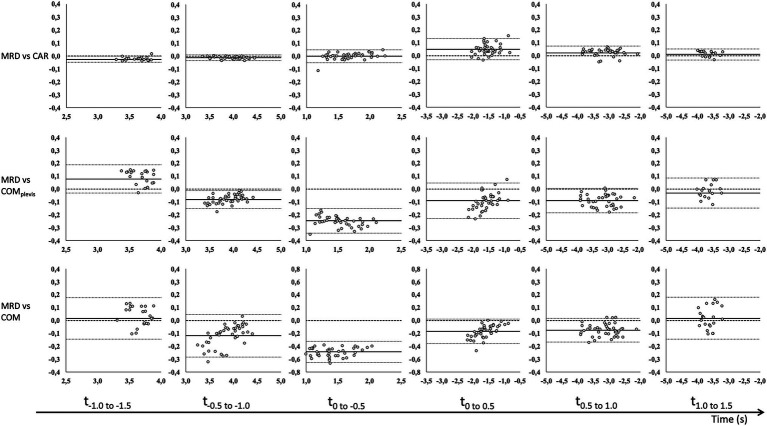
Bland–Altman analysis of average velocity for 0.5 s time intervals before (−) and after (+) CoD (horizontal axis) for the MRD to CAR (top row), COM_pelvis_ (middle row) and COM (bottom row) for 9 kg external load. Bland–Altman plots [y-axis: difference in velocity (m/s) and x-axis: average velocity (m/s)] for all trials with fixed bias (full line) with 95% confidence interval (dotted line) and agreement (dashed line).

Correlations between MRD_vel_ and CAR_vel_, COM_pelvis_vel_, and COM_vel_ were mostly very good to excellent. Specifically, correlation coefficients between MRD_vel_ and the other outcome variables ranged from 0.93 to 1.00, 0.53 to 1.00 and 0.93 to 1.00 for 3, 6, and 9 kg external load, respectively (Table 2). CV values ranged from 0.3 to 3.2%, 0.8 to 4.3%, and 1.5 to 7.7% for the CAR_vel_, COM_pelvis_vel_, and COM_vel_ comparisons. The observed biases for CAR_vel_ comparisons ranged from −0.027 to 0.05 m/s, −0.246 to 0.128 m/s, and −0.486 to 0.082 m/s across all loaded conditions and time intervals for the CAR_vel_, COM_pelvis_vel_, and COM_vel_ comparisons, respectively (Table 2). Bland–Altman analyses for the same time intervals (0.5 s) are presented in Figures 2–4 for the 3, 6, and 9 kg conditions, respectively.

**Table 2:** Average velocity and criterion validity of a motorized resistance device for different time intervals before (−) and after (+) CoD.

		Average velocity (M ± SD)				Criterion validity (MRD_vel_ to CAR_vel_ comparison)				Criterion validity (MRD_vel_ to COM_pelvis_vel_ comparison)				Criterion validity (MRD_vel_ to COM_vel_ comparison)			
Load (kg)	Interval	MRD_vel_	CAR_vel_	COM_pelvis_vel_	COM_vel_	Bias (CI; LOA)	Correlation	CV (95 CI)	n/n_tot_	Bias (CI; LOA)	Correlation	CV (95 CI)	n/n_tot_	Bias (CI LOA)	Correlation	CV (CI)	n/n_tot_
3	−1.0 to −1.5	3.39 ± 0.26	3.36 ± 0.25	3.52 ± 0.24	3.45 ± 0.23	−0.023 (−0.055; 0.010)	*r* = 1.00	0.5 (0.4; 0.7)	24/40	0.128 (0.006; 0.25)	*r* = 0.97	1.7 (1.3; 2.3)	23/40	0.072 (−0.073; 0.22)	*r* = 0.96	1.9 (1.5; 2.6)	23/40
	−0.5 to −1.0	4.03 ± 0.24	4.02 ± 0.23	3.96 ± 0.27	3.94 ± 0.27	−0.012 (−0.04; 0.02)	*r* = 1.00	0.4 (0.3; 0.4)	40/40	−0.064 (−0.18; 0.050)	*r* = 0.98	1.4 (1.2; 1.8)	39/40	−0.090 (−0.22; 0.038)	*r* = 0.98	1.5 (1.3; 1.9)	39/40
	0 to −0.5	1.92 ± 0.35	1.95 ± 0.36	1.69 ± 0.33	1.50 ± 0.33	0.026 (−0.023; 0.076)	*r* = 1.00	1.3 (1.1; 1.7)	40/40	−0.23 (−0.33; 0.13)	*r* = 0.99	2.9 (2.4; 3.5)	39/40	−0.435 (−0.64; −0.23)	*r* = 0.96	7.4 (6.2; 9.3)	39/40
	0 to 0.5	−1.77 ± 0.26	−1.73 ± 0.26	−1.89 ± 0.28	−2.03 ± 0.26	0.039 (0.036; 0.11)	*r* = 0.99	2.3 (2.0; 2.9)	40/40	−0.126 (−0.25; 0.00)	*r* = 0.97	3.7 (3.1; 4.6)	39/40	−0.259 (−0.44; −0.074)	*r* = 0.93	5.1 (4.3; 6.3)	39/40
	0.5 to 1.0	−3.68 ± 0.38	−3.65 ± 0.38	−3.78 ± 0.39	−3.77 ± 0.40	0.032 (0.0027; 0.061)	*ρ* = 1.00	0.4 (0.3; 0.5)	40/40	−0.094 (−0.19; 0.005)	*r* = 0.99	1.4 (1.2; 1.8)	39/40	−0.079 (−0.19; 0.03)	*ρ* = 0.98	1.6 (1.3; 1.9)	39/40
	1.0 to 1.5	−3.95 ± 0.43	−3.95 ± 0.40	−3.88 ± 0.45	−3.83 ± 0.44	−0.004 (−0.072; 0.064)	*r* = 1.00	0.7 (0.5; 1.2)	9/40	0.042 (−0.13; 0.22)	*r* = 0.98	2.6 (1.8; 5.0)	8/40	0.082 (−0.055; 0.22)	*r* = 0.99	2.1 (1.4; 4.0)	8/40
6	−1.0 to −1.5	3.53 ± 0.22	3.50 ± 0.22	3.62 ± 0.20	3.55 ± 0.18	−0.029 (−0.048; −0.01)	*ρ* = 0.99	0.3 (0.2; 0.4)	18/40	0.097 (0.002; 0.191)	*r* = 0.98	1.0 (0.8; 1.5)	17/40	0.022 (−0.139; 0.183)	*r* = 0.93	1.9 (1.5; 2.8)	18/40
	−0.5 to −1.0	4.03 ± 0.29	4.02 ± 0.28	3.98 ± 0.30	3.96 ± 0.31	−0.016 (−0.036; 0.005)	*r* = 1.00	0.2 (0.2; 0.3)	38/40	−0.065 (−0.143; 0.040)	*r* = 0.99	1.0 (0.8; 1.2)	36/40	−0.075 (−0.207; 0.058)	*r* = 0.98	1.7 (1.4; 2.1)	38/40
	0 to −0.5	1.92 ± 0.27	1.94 ± 0.29	1.68 ± 0.25	1.46 ± 0.29	0.017 (−0.019; 0.053)	*r* = 1.00	0.8 (0.7; 1.0)	39/40	−0.241 (−0.321; −0.161)	*r* = 0.99	2.3 (1.9; 2.9)	36/40	−0.461 (−0.627; −0.295)	*r* = 0.96	6.5 (5.5; 8.2)	39/40
	0 to 0.5	−1.65 ± 0.24	−1.62 ± 0.24	−1.77 ± 0.26	−1.91 ± 0.26	0.037 (−0.022; 0.095)	*r* = 1.00	2.0 (1.7; 2.5)	39/40	−0.120 (−0.225; −0.014)	*r* = 0.98	3.0 (2.5; 3.8)	36/40	−0.252 (−0.406; −0.098)	*r* = 0.96	4.3 (3.6; 5.3)	39/40
	0.5 to 1.0	−3.41 ± 0.42	−3.38 ± 0.42	−3.48 ± 0.43	−3.47 ± 0.42	0.034 (0.004; 0.064)	*ρ* = 1.00	0.5 (0.4; 0.6)	39/40	−0.075 (−0.154; 0.004)	*ρ* = 0.98	1.2 (1.0; 1.6)	36/40	−0.062 (−0.147; 0.023)	*ρ* = 0.98	1.3 (1.1; 1.6)	39/40
	1.0 to 1.5	−3.88 ± 0.19	−3.87 ± 0.19	−3.87 ± 0.25	−3.82 ± 0.22	0.005 (−0.054; 0.064)	*r* = 0.99	0.8 (0.6; 1.2)	13/40	−0.005 (−0.170; 0.159)	*ρ* = 0.89	2.1 (1.5; 3.3)	12/40	0.061 (−0.156; 0.277)	*ρ* = 0.53	3.0 (2.2; 4.7)	13/40
9	−1.0 to −1.5	3.75 ± 0.24	3.72 ± 0.24	3.82 ± 0.22	3.77 ± 0.22	−0.027 (−0.050; −0.003)	*r* = 1.00	0.3 (0.3; 0.4)	29/44	0.077 (−0.032; 0.186)	*r* = 0.98	1.2 (1.0; 1.5)	28/44	0.015 (−0.146; 0.177)	*r* = 0.94	2.0 (1.6; 2.5)	29/44
	−0.5 to −1.0	3.91 ± 0.32	3.90 ± 0.32	3.83 ± 0.33	3.79 ± 0.38	−0.012 (−0.033; 0.009)	*r* = 1.00	0.3 (0.2; 0.3)	44/44	−0.081 (−0.150; −0.012)	*r* = 1.00	0.8 (0.7; 1.0)	42/44	−0.118 (−0.284; 0.047)	*r* = 0.98	2.0 (1.7; 2.4)	44/44
	0 to −0.5	1.61 ± 0.34	1.61 ± 0.35	1.36 ± 0.32	1.12 ± 0.33	−0.001 (−0.050; 0.048)	*r* = 1.00	1.8 (1.5; 2.2)	44/44	−0.246 (−0.341; −0.150)	*r* = 0.99	3.5 (2.9; 4.3)	42/44	−0.486 (−0.650; −0.322)	*r* = 0.97	7.7 (6.5; 9.5)	44/44
	0 to 0.5	−1.51 ± 0.28	−1.46 ± 0.29	−1.59 ± 0.33	−1.68 ± 0.34	0.050 (−0.031; 0.131)	*r* = 0.99	3.2 (2.7; 3.9)	44/44	−0.090 (−0.227; 0.047)	*r* = 0.98	4.3 (3.6; 5.3)	42/44	−0.170 (−0.358; 0.019)	*r* = 0.97	4.9 (4.1; 6.0)	44/44
	0.5 to 1.0	−3.08 ± 0.43	−3.05 ± 0.43	−3.15 ± 0.43	−3.15 ± 0.43	0.022 (−0.030; 0.075)	*r* = 1.00	0.9 (0.8; 1.1)	43/44	−0.089 (−0.183; 0.004)	*r* = 0.99	1.6 (1.3; 1.9)	41/44	−0.075 (−0.166; 0.017)	*r* = 0.99	1.5 (1.3; 1.9)	43/44
	1.0 to 1.5	−3.63 ± 0.22	−3.62 ± 0.21	−3.65 ± 0.22	−3.62 ± 0.23	0.009 (−0.033; 0.051)	*r* = 1.00	0.6 (0.5; 0.8)	22/44	−0.031 (−0.148; 0.085)	*r* = 0.96	1.7 (1.4; 2.4)	21/44	0.017 (−0.146; 0.179)	*r* = 0.93	2.4 (1.9; 3.3)	22/44

MRD_vel_, velocity measured by motorized technology; CAR_vel_, velocity of marker placed on carabiner; COM_pelvis_vel_, velocity of center of mass pelvis; COM_vel_, velocity of center of mass; M, mean; SD, standard deviation; CI, Confidence interval; LOA, limits of agreement; r, Pearson correlation coefficient; *ρ*, Spearman rank-order correlation coefficient; CV, Coefficient of variation; n, trials analyzed; n_tot_, total number of trials.

**Figure 5:**
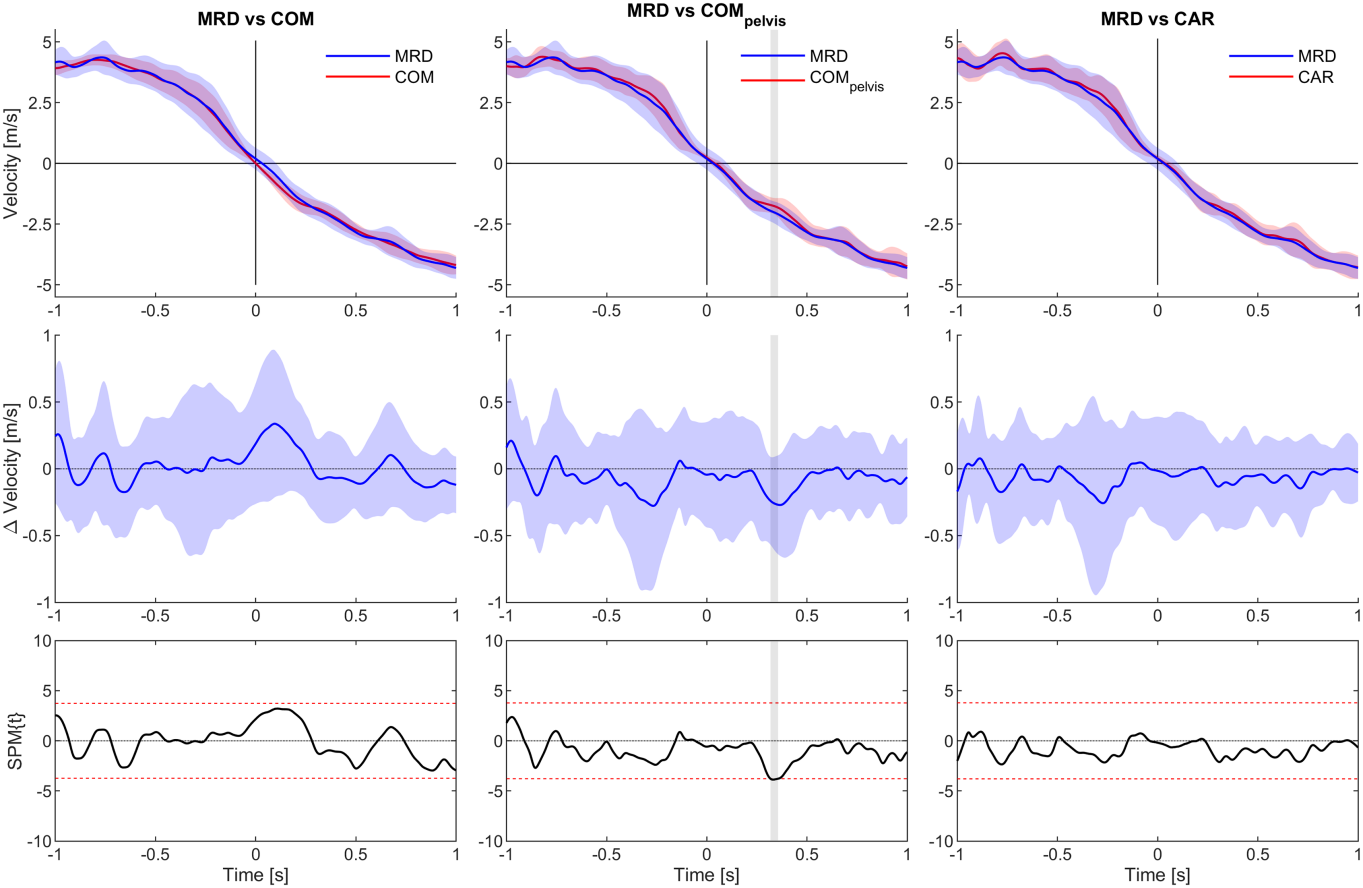
SPM analysis of average velocity for 1.0 s time interval before (−) and after (+) CoD (horizontal axis all graphs) for the 3 kg condition. Columns left to right show MRD to COM, MRD to COM_pelvis_ and MRD to CAR comparisons. Top row show average velocity of all trials with 95% confidence interval for the MRD (blue) to COM, COM_pelvis_ and CAR (red) comparisons. Middle row show difference in velocity with 95% confidence interval. Bottom row show SPM analysis with 95% confidence interval marked with red dashed lines. Grey vertical line identify time interval of significant difference (*α* < 0.05).

**Figure 6:**
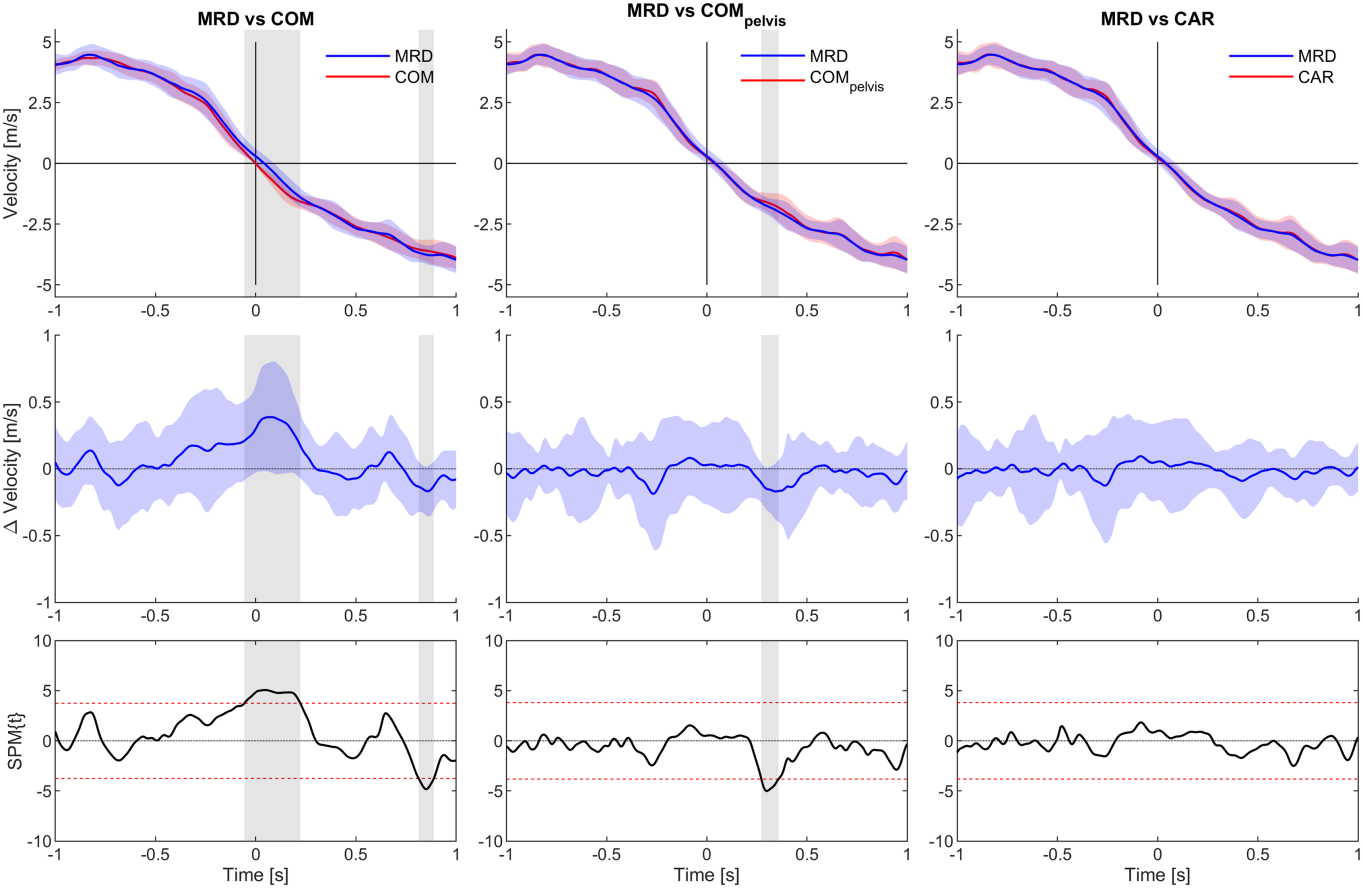
SPM analysis of average velocity for 1.0 s time interval before (−) and after (+) CoD (horizontal axis all graphs) for the 6 kg condition. Columns left to right show MRD to COM, MRD to COM_pelvis_ and MRD to CAR comparisons. Top row show average velocity of all trials with 95% confidence interval for the MRD (blue) to COM, COM_pelvis_ and CAR (red) comparisons. Middle row show difference in velocity with 95% confidence interval. Bottom row show SPM analysis with 95% confidence interval marked with red dashed lines. Grey vertical line identify time interval of significant difference (*α* < 0.05).

**Figure 7:**
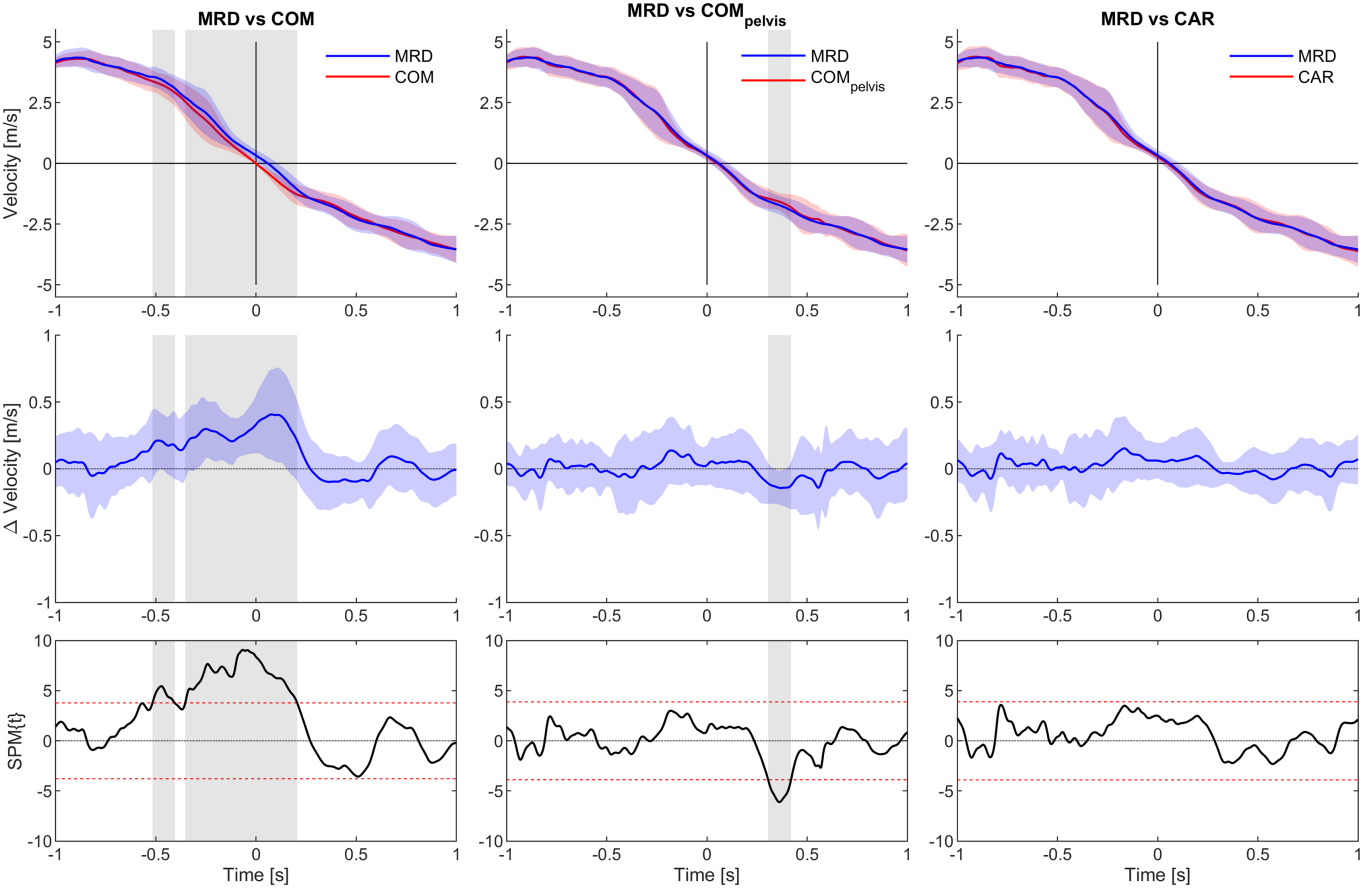
SPM analysis of average velocity for 1.0 s time interval before (−) and after (+) CoD (horizontal axis all graphs) for the 9 kg condition. Columns left to right show MRD to COM, MRD to COM_pelvis_ and MRD to CAR comparisons. Top row show average velocity of all trials with 95% confidence interval for the MRD (blue) to COM, COM_pelvis_ and CAR (red) comparisons. Middle row show difference in velocity with 95% confidence interval. Bottom row show SPM analysis with 95% confidence interval marked with red dashed lines. Grey vertical line identify time interval of significant difference (*α* < 0.05).

Statistical parametric mapping analysis yielded no significant difference between MRD_vel_ and CAR_vel_ for the any of the load conditions. However, significant underestimation from 0.32 to 0.36 s for the COM_pelvis_vel_ comparison was observed for the 3 kg load condition. For the 6 kg load condition, significant overestimation from −0.06 to 0.22 s and underestimation from 0.81 to 0.89 s for the COM_vel_ comparison was observed, while a significant underestimation from 0.27 to 0.36 s for the COM_pelvis_vel_ comparison. For the 9 kg load condition, significant overestimation from −0.52 to −0.41 and from −0.35 to 0.21 s for the COM_vel_ comparison were observed, while a significant underestimation from 0.31 to 0.42 s interval for the COM_pelvis_vel_ comparison (Figures 5–7).

## Discussion

The aim of this study was to explore validity of velocity measurements obtained from a MRD under different loaded conditions during a m505 test. The MRD provided valid velocity measurements as excellent correlation coefficients and CV values indicate a close relationship between motion capture data and MRD_vel_ in each phase of the CoD. Furthermore, this excellent relationship is maintained for the different loaded conditions. The observed biases differ when MRD_vel_ is compared to CAR_vel_, COM_pelvis_vel_, and COM_vel_ for the different time intervals. Specifically, the MRD_vel_ to CAR_vel_ comparison yielded small observed biases for all time intervals, which increased for the COM_pelvis_vel_ and COM_vel_ comparison.

The same MRD has been compared to timing gate measurements previously in sprint running (Rakovic et al., 2022). The outcome measurements in that study were 0–30 m split time measurements with fair to excellent correlations (0.48–0.95). Overall, better correlations were observed in the current study during a m505 task as they were excellent with one exception (Table 2). The excellent correlations highlight the strong relationship between the different outcome variables for the different time intervals, but do not provide information about agreement, which was explored using Bland–Altman analysis. The smallest biases were observed between MRD_vel_ and CAR_vel_ for all time intervals captured during the CoD. The same MRD yielded small biases and was within the limits of precision of ±0.01 s for 5–30 m linear sprint split times, with exception being the 0–5 m interval which is explained by onset of measurement (Rakovic et al., 2022). If the average 5-min split times (5–20 m) from that study is converted to velocity, the observed velocity bias range from 0.011 to 0.082 m/s (Rakovic et al., 2022). Based on these values, the observed biases in the current study for the CAR_vel_ comparisons are smaller, the COM_pelvis_vel_ comparisons comparable, while the COM_vel_ comparisons have greater biases. However, it is important to note that split time velocity measurements in a linear sprint are greater, which makes the relative values smaller. The greater observed biases for the COM_pelvis_vel_ and COM_vel_ comparisons can be explained by the fact that they are based on calculations from multiple markers on the pelvis and whole body, respectively. Especially, COM_vel_ calculations are subject to both upper and lower extremity movements, which are not measured by the MRD, as the point of attachment to the subject is on a pelvic belt. However, the valid velocity measurements of both a single point (CAR_vel_) and pelvis (COM_pelvis_vel_) are useful. In fact, in linear sprint both laser and radar are accepted measures of athlete movement and used for validation purposes (Morin et al., 2019). Laser measurements of linear sprint are based on one point, or a moving point, on the backside of the athlete performing the sprint that has proven useful in linear sprint assessments. This procedure is similar to the CAR_vel_ measurement in the current study.

Obviously external load will impact overall CoD test performance time through providing “assistance” and “resistance” during different phases of the CoD. For example, during the initial acceleration-to-deceleration phase (phase 1a) and due to the positioning of the MRD, the athlete is “assisted” as they accelerate and then decelerate prior to CoD. In contrast, during the re-acceleration phase (phase 1b) the athlete is faced with a resisted load that would essentially reduce whole body acceleration, especially with increasing external load (i.e., 3–9 kg). Indeed, in the current study overall time went from 3.26 s with 3 kg, to 3.32 s for 6 kg, to 3.52 s for the 9 kg external load. Furthermore, the observed standard deviations for overall time for the different load conditions ranged from 0.29 to 0.35 s indicating a fairly wide distribution of performances on the m505 resisted test. This ensures validity over a greater range of performances. Furthermore, the inclusion of both males and females as well as different sports (soccer, basketball, handball, tennis, and floorball) improves validity to encompass different ball sports and gender. However, phase-specific comparisons of measurements (time, distance, and average velocity) have not been done as the authors are unaware of such information being reported elsewhere.

Phase-specific information obtained during a 45 and 90° CoD test has previously been explored using two synchronized laser guns (Hader et al., 2015). However, only overall time was validated against timing gates, while reliability analysis included peak and distance-to-peak acceleration, deceleration, and speed, along with minimum speed, and speed between 8 and 12 m that represented the distance 2 m prior and 2 m after the CoD. The CV values for speed around the 8–12 m distance were ~5%, and for phase-specific information CV values ranged from 6.6 to 8.5% for peak acceleration, and from 117 to 12.6% for peak and distance-to-peak deceleration. In the current validity study, CV values for average velocity measurements for the different time intervals ranged from 0.3 to 7.7% for all comparisons. Overall, these CV values are smaller than those reported by Hader and co-author, but it is important to emphasize that our CV values represent phase-specific validity of velocity measurements, while those reported by Hader and co-workers represent phase-specific reliability of different variables computed from meter-to-meter changes in speed over time recorded at 100 Hz. In the current study, continuous velocity measurements recorded at 333 Hz were analyzed, which might be more suitable to obtain rapid acceleration and deceleration data during CoD. Furthermore, the correlations of overall time reported by Hader and co-authors, which they used for validity analysis, are similar to those observed in the current study.

Motion capture data for the full m505 test would have allowed for more comparisons to MRD_vel_, especially the 1.5 to 1.0 s interval before and after the CoD (Table 2). Trials included for the time intervals 1 s prior to and after the CoD included a large percentage of total tests done and ranged from 38 to 44 tests. However, for the time intervals 1.5 to 1.0 s before and after CoD the number of tests analyzed ranged from 8 to 22 tests. The reason for this lower number of tests was quality of kinematic data. The recording volume included the start and end position of the m505 test, but there were challenges getting good quality kinematic data of markers necessary for the analysis. This might be due to fewer cameras being able to observe all markers at the start and the end of the test. Furthermore, the above is also the reason why the SPM analysis was employed for 1 s before and after CoD. In addition, the reason for selecting 1.5 s pre- and post-CoD was that others have found total m505 test times to be in the range of 2–3 s (Nimphius et al., 2017). The above in combination with not removing any outliers (Hoaglin et al., 1986) might explain why moderate correlation (*ρ* = 0.53) was observed for the MRD_vel_ to COM_vel_ comparison for the t_1.0–1.5_ time interval with 6 kg external load.

Furthermore, MRD_vel_ and kinematic data were synchronized by cross-correlation in post-processing, which may have impacted our results. However, from the mostly excellent correlations, small CV values and biases for the CAR_vel_ as well as for the COM_vel_ and COM_pelvis_vel_ comparison it appears that the continuous velocity measurements obtained by the MRD is a valid representation of subject velocity during the different phases of a m505 test. Since the m505 is performed across short distances (i.e., 5 m in and out of CoD), future research should look to examine the validity of the motorized device when entry velocity on approach to CoD and deceleration demands are higher.

The impulse–momentum relationship dictates that the ability to generate horizontal forces during both initial acceleration-to-deceleration and re-acceleration phases are key to CoD performance. During deceleration, horizontally oriented braking forces are important to performance of 505 CoD tests (Dos’Santos et al., 2020), while during acceleration the horizontal propulsive component of the ground reaction force is important to performance (Morin et al., 2011). Based on these findings and the high horizontal deceleration and acceleration demands in team sports (Harper et al., 2019), providing horizontal load prescription for training to improve CoD ability could be of considerable importance. Specifically, external load provided by motorized resistance will provide “assistance” and “resistance” during different phases of the CoD. Consequently, the application of horizontal loading with different resisted or assisted loads and how the athlete starts (moving away or toward the device) might further improve not only testing, but also training strategies to target CoD ability.

## Conclusion

The velocity measurements obtained by a MRD during a m505 test are valid when compared to three-dimensional motion analysis data. Validity analyses yielded low biases and CV values with excellent correlations for the MRD_vel_ to CAR_vel_ comparison. The increased observed biases and lower CV values for the COM_vel_ and COM_pelvis_vel_ comparisons are to be expected as the MRD represent movement of the point of attachment to the athlete, especially for the COM_vel_ comparison as the kinematic method used quantifies upper and lower extremity movements during the test. As single points of measurement (i.e., laser) are useful to assess other athletic tasks (i.e., sprint running), the single point CAR_vel_ comparison is appropriate for the m505 test. Thus, velocity measurements obtained from a MRD during the m505 test provide researchers and coaches alike with new opportunities to advance assessment and understanding of their athletes CoD abilities.

## Practical Applications

Our findings have the potential to influence not only field, but also lab-based testing and training of CoD. Continuous and phase-specific information (time, distance, and average velocity) can provide coaches with important information that previously only was available in a lab setting. In turn, such information can be used to target specific CoD phases in training with the use of horizontal loading. Furthermore, how velocity changes during a m505 tests may allow for calculation of change of momentum during the test and thereby increase our understanding of this important quality in a much more time efficient manner (Nimphius et al., 2017). In addition, the MRD used in the current study provides opportunity to explore athletes deceleration performance during CoD in more detail than was previously possible, similar to methods described using a radar device during a horizontal acceleration-to-deceleration test (Harper et al., 2020).

## Data Availability Statement

The raw data supporting the conclusions of this article will be made available by the authors, without undue reservation.

## Ethics Statement

The studies involving human participants were reviewed and approved by Norwegian School of Sport Sciences. Written informed consent to participate in this study was provided by the participants’ legal guardian/next of kin.

## Author Contributions

OE, FA, DH, and ØG contributed to the data collection, interpretation, and revising the manuscript. The original study design was made by OE and ØG and discussed with FA.

## Conflict of Interest

OE is a shareholder in 1080 Motion AB and 1080 MAP AS.

The remaining authors declare that the research was conducted in the absence of any commercial or financial relationships that could be construed as a potential conflict of interest.

## Publisher’s Note

All claims expressed in this article are solely those of the authors and do not necessarily represent those of their affiliated organizations, or those of the publisher, the editors and the reviewers. Any product that may be evaluated in this article, or claim that may be made by its manufacturer, is not guaranteed or endorsed by the publisher.
